# Prevalence, Detection, and Management of the Metabolic Syndrome in Patients with Acute Myocardial Infarction: Role of an Obesity-Centric Definition

**DOI:** 10.4061/2010/814561

**Published:** 2010-07-27

**Authors:** Sandhir B. Prasad, Farzan Fahrtash, Yuvaraj Malaiapan, Ian T. Meredith, James Cameron

**Affiliations:** Monash Heart and Monash Cardiovascular Research Centre, Monash Medical Centre, 246 Clayton Road, Clayton, Melbourne, Victoria 3168, Australia

## Abstract

*Background*. We sought to determine and compare the prevalence of the Metabolic Syndrome (MS) in patients with acute myocardial infarction (AMI) utilizing the new International Diabetes Federation (IDF) definition with the older National Cholesterol Education Program (NCEP) definition. We also examined the clinical utility of MS in this context. *Methods*. A total of 107 consecutive patients with AMI were prospectively evaluated for MS. Fasting lipids obtained at admission and fasting glucose at discharge were used. A postdischarge folder audit verified rates of discharge coding and implementation of specific management strategies for MS. *Results*. Baseline patient characteristics included: mean age 59 ± 13 years; males 80%; diabetes 19%; mean BMI 29.7 ± 8.4 kg/m^2^. MS prevalence was 54% by the IDF definition and 49% by the NCEP definition, with good agreement between definitions: *κ* = 0.664, *P* < .001. Factors predictive of MS after multivariate analysis included: hypertension, fasting glucose, waist circumference, and serum HDL (all *P* < .05). Despite the high prevalence, MS was recognized at discharge in only 1 patient, and referral for exercise and/or weight-loss programs was undertaken in 5 patients. *Conclusion*. There is a high prevalence of MS utilizing contemporary definitions in patients with AMI: 54% by the IDF definition and 49% by NCEP criteria. Despite the high prevalence, MS was under-recognized and under-treated in this population.

## 1. Introduction

The metabolic syndrome (MS) has been shown to be a powerful and potentially modifiable risk factor for coronary artery disease as well as diabetes [1–3]. There is limited data on the prevalence of MS in patients with acute myocardial infarction (AMI) utilizing contemporary definitions [4]. In particular, no study has previously reported the prevalence of MS in this population using the International Diabetes Federation (IDF) definition, which critically differs from previous definitions in that it emphasizes central obesity as the essential criterion for MS diagnosis [5]. In addition, no previous study has focused on the clinical utility of MS to physicians in the routine management of patients with AMI.

The aim of the present study was to determine and compare the true prevalence of MS in patients with AMI utilizing the new “obesity-centric” IDF definition with the older National Cholesterol Education Program (NCEP) definition. We also sought to examine the clinical utility of the MS concept in this context by reviewing rates of clinical diagnosis and institution of specific management strategies for MS by clinicians managing patients with AMI. We hypothesized that, given the composite definition of MS incorporating well-recognized cardiac risk factors, a high prevalence of MS would be noted in this population, and that rates of clinical detection would be low owing to variation in definitions and controversy about etiology.

## 2. Materials and Methods

We conducted a prospective observational study involving 107 consecutive patients with acute ST-elevation myocardial infarction (STEMI) admitted to the coronary care unit of a regional tertiary referral center between May and November 2007. All patients were referred for primary percutaneous coronary intervention (PCI) and had prompt coronary angiography as per protocol. Fasting lipid assays (total cholesterol (T-Chol), high-density lipoprotein cholesterol (HDL-C), low-density lipoprotein cholesterol (LDL-C), triglycerides (Trig)) were performed the morning after admission (within 24 hours of admission). Fasting glucose was performed on day 4 post admission or day prior to discharge whichever was later. Patients were reviewed on the ward and anthropometric measurements including waist circumference, hip circumference, height, and weight were obtained on day of discharge. All patients were referred for echocardiography prior to discharge as per ward protocol. The presence of the MS was determined according to the two most contemporary definitions: the International Diabetes Federation (IDF) consensus definition (2006) and the National Cholesterol Education Panel (NCEP) definition (2001) (see below for definitions). Following discharge of patients from the ward, a folder audit was carried out to determine rates of discharge diagnosis coding of MS. Specific aspects of the recommended management of MS were also audited, namely, referral for dietary advice, exercise programs, and/or weight loss programs. All data collection was performed by researchers independent of the clinical teams involved in patient care. All blood assay results were available to the clinical teams involved in patient care, and internists were alerted to abnormal fasting glucose levels for referral to inpatient diabetes services. Angiographic and echocardiographic data are reported as documented in the final reports produced by the reporting cardiologist blinded to MS status.

Serum lipid measurements were made from 5 mls of blood collected in a SST gel vacutainer. Serum glucose measurements were made from 2 mls of blood collected in a Sodium Fluoride EDTA vacutainer. All serum assays were performed on a Beckman-Coulter Unicel DxC800 analyzer (Fullerton, California, USA). Glucose measurements were obtained enzymatically using the glucose oxidase method. T-Chol and HDL-C measurements were obtained enzymatically using the cholesterol oxidase method. Triglyceride (Trig) measurements were obtained enzymatically using glycerol kinase and glycerophosphate oxidase method. LDL-C levels were derived using the formula: LDL-C = T-Chol – HDL-C – Trig/2.25. 

### 2.1. Definitions

MS according to the IDF definition (IDF-MS) was defined as per current guidelines as having ethnic-specific waist circumference (WC) cutoff values as the measure of central obesity, plus any two of raised triglycerides ≥150 mg/dL (1.7 mmol/L) or specific treatment for this abnormality, reduced high-density lipoprotein cholesterol (HDL-cholesterol) <40 mg/dL (1.03 mmol/L) in men and <50 mg/dL (1.29 mmol/L) in women or specific treatment for this abnormality, raised blood pressure (BP) ≥130 mmHg systolic and ≥85 mmHg diastolic or treatment of previously diagnosed hypertension, and raised fasting plasma glucose (FPG) ≥100 mg/dL (5.6 mmol/L) or previously diagnosed diabetes [6].

The metabolic syndrome according to the NCEP definition (NCEP-MS) was defined as per current guidelines as having a cluster of at least 3 of the following characteristics: fasting glucose ≥110 mg/dL (6.0 mmol/L) or taking medications for diabetes, central obesity with WC >102 cm in men and >88 cm in women, raised triglycerides ≥150 mg/dL (1.7 mmol/L), low HDL-cholesterol < 40 mg/dL (1.03 mmol/L) in men and <50 mg/dL (1.29 mmol/L) in women, or raised BP ≥135 mmHg systolic and ≥85 mmHg diastolic or previously diagnosed hypertension [7].

Those with a preadmission diagnosis of diabetes were considered to have established diabetes. New impaired fasting glucose (IFG) was defined as a predischarge fasting plasma glucose of ≥100 mg/dL (5.6 mmol/L) in patients without pre-existing diabetes [8]. Obesity was defined as a body-mass index (BMI) ≥30 kg/m^2^ [9]. Hypercholesterolemia was defined as fasting serum total cholesterol of ≥4.5 mmol/L [7]. ECG criteria for STEMI was in accordance with currently accepted guidelines: ST elevation ≥1 mm in two contiguous limb leads, ≥2 mm in two contiguous chest leads, or new onset left bundle branch block (LBBB) [10]. Significant coronary disease was defined as luminal narrowing of ≥50% on angiography

### 2.2. Statistical Analysis

Data was analysed using SPSS for Windows version 14.0 (SPSS Inc, Chicago, Illinois). Categorical variables are presented as percentages and continuous variables as mean ± standard deviation. Group differences were assessed using Student's *t*-test for continuous variables and Fisher's exact test for categorical variables. Agreement between the 2 definitions of MS was assessed using Cohen's Kappa (*κ*). Logistic regression analysis with stepwise selection with inclusion of variables at the 5% level and removal at the 10% level at each step was used to identify independent predictors of MS from baseline patient characteristics and laboratory findings. Both forwards and backwards selection were used to arrive at the same model. A *P* value <.05 was considered statistically significant.

### 2.3. Ethical Considerations

This study was approved by the institutional ethical review committee, and informed consent was obtained from each patient before participation.

## 3. Results

The baseline patient characteristics of the 107 patients enrolled in this study are listed in Table 1. The mean age was 59 ± 13 years, and 80% were males. The predominant ethnic grouping was Europid (*n* = 79; 74%). Pre-existing hypertension was the most common cardiac risk factor, found in 51 patients (48%). Pre-existing diabetes was present in 20 patients (19%). New IFG in patients without pre-existing diabetes was found in 42 patients (39%). Baseline anthropometric measurements suggested an overweight population with a mean BMI of 29.7 ± 8.4 kg/m^2^. Baseline serum lipid measurements on day 1 of admission were total cholesterol 4.7 ± 1.3 mmol/L, HDL 1.0 ± 0.3 mol/L, LDL 3.0 ± 1.1 mmol/L, triglycerides 1.5 ± 0.8 mmol/L.

A total of 58 patients (54%) met the criteria for IDF MS. A comparison of patients with and without IDF MS is presented in Table 2. Significant differences on univariate analysis were noted between the 2 groups in the prevalence of hypertension, pre-existing diabetes, and new IFG in terms of clinical characteristics; waist circumference, weight, and BMI in terms of anthropometric measurements; fasting glucose, HDL, and triglycerides in term of laboratory measurements. While no differences were noted between the 2 groups in terms of systolic function measured by the left ventricular ejection fraction, a shorter deceleration time (DT) (DT 174.0 ± 61.7 versus 206.0 ± 58.0 ms, *P* = .02) on Doppler echocardiography and a lower E′ velocity on tissue Doppler (5.2 ± 1.6 m/s and 6.5 ± 2.0 m/s, *P* = .04) suggested greater diastolic impairment in the MS group. There were no differences between the 2 groups in the extent of coronary disease and coronary flow post PCI.

A total of 52 patients (49%) met the criteria for NCEP MS. A comparison of patients with and without NCEP MS is presented in Table 3. Significant differences on univariate analysis were noted between the 2 groups in terms of age, gender, hypertension new IFG in terms of clinical characteristics; waist circumference and weight in terms of anthropometric measurements; fasting glucose, HDL and triglycerides in terms of laboratory measurements. While no differences were noted between the 2 groups in terms of systolic function measured by the left ventricular ejection fraction, indices of diastolic function assessed by tissue Doppler suggested greater diastolic impairment in the MS group: E′ velocity 5.4 ± 1.6 versus 6.3 ± 1.9 m/s, *P* = .046 and E to E′ ratio 14.8 ± 5.5 versus 11.8 ± 4.1, *P* = .014. There were no differences between the 2 groups in the extent of coronary disease and coronary flow post PCI.

**Figure 1:**
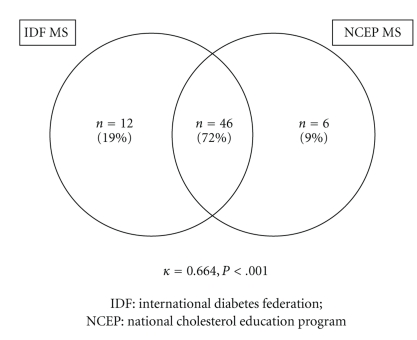
Prevalence of the metabolic syndrome according to different definitions.

The agreement between definitions for the presence of MS is illustrated in Figure 1. Out of the 64 patients (60%) who had MS by either definition, 46 patients (72%) had MS by both definitions, 12 patients (19%) had MS by the IDF definition only, and 6 patients (9%) had MS by the NCEP definition only. The number of patients without MS by either definition was 43 (40%). Cohen's Kappa for the agreement between the 2 definitions for the presence of MS was *κ* = 0.664, *P* < .001, suggestive of a moderately high degree of agreement between these two contemporary definitions.

A comparison of the number of features of MS other than waist circumference in patients with and without central obesity as defined by the obesity centric IDF definition showed that patients who exceeded the waist circumference cutoffs (*n* = 85; 79%) had significantly higher number of other features (2.2 ± 1.2 versus 1.5 ± 1.2, *P* = .028) of MS, suggesting a role for causation linked to central obesity. From these 85 patients (79%) who exceeded the IDF waist circumference cutoff, 58 (68%) satisfied the criteria for IDF MS.

Multivariate predictors of MS are presented in Table 4. For NCEP MS, hypertension, serum fasting glucose, serum triglyceride levels, and waist circumference remained predictive of MS. Serum HDL levels and male gender were inversely related to the presence of MS. For IDF MS, hypertension, waist circumference and serum fasting glucose remained predictive of MS, while serum HDL levels were inversely related.

The results of the postdischarge folder audit showed that while 64 patients (60%) had MS by either definition, it was clinically recognized on discharge in only 1 patient (1%). Referral for exercise or weight loss programs was undertaken in 5 patients (5%) only. While all patients received brief generic dietary advice following their AMI, specific referral to a dietitian was only undertaken in 17 patients (16%).

## 4. Discussion

The present study provides a first assessment of the prevalence of MS in patients with AMI using the “obesity-centric” IDF definition and compares it to the prevalence using the more traditional NCEP definition. The main finding of this study is that there is a high prevalence of MS using these contemporary definitions in patients with AMI: 54% according to the 2005 IDF definition and 49% according to the 2001 NCEP definition. The prevalence of MS (by either definition) was greater than any other standard cardiac risk factor in this population. There was a high degree of concordance between the obesity-centric IDF and the nonobesity centric NCEP definitions for the diagnosis of MS (*κ* = 0.664, *P* < .001). However, despite the markedly high prevalence of MS in this high-risk population, there was a low rate of clinical detection of MS by clinicians, raising questions about the clinical utility of this syndrome in this context.

Evolving understanding of MS has led to multiple definitions over the years, with variable emphasis on the role of insulin resistance in the pathophysiology of MS [1, 2, 5]. The NCEP definition, for example, places equal emphasis on all of the components comprising the cluster of metabolic derangements rather than emphasizing either insulin resistance or obesity [2, 11]. The IDF definition, by contrast, is the first to emphasize the measure of central obesity over insulin resistance as the essential criterion for MS diagnosis, and as such draws a vital connection between the modern “obesity epidemic” and the metabolic derangements seen in MS [5]. This represents an important shift in the current understanding of MS, and has important implications for both primary and secondary prevention of coronary disease with respect to both obesity and MS. In the current study, despite the relatively small sample size, the number of features of MS other than central obesity was noted to be higher in patients who exceeded the waist circumference cutoffs (2.2 ± 1.2 versus 1.5 ± 1.2, *P* = .028), confirming a potential causative role for central obesity. Also, given that all patients with IDF MS had obesity by definition, presence of these additional MS-related metabolic derangements may be a way of refining risk assessment in obese patients.

The diagnosis of MS following an AMI poses several challenges. The physiological and psychological stress associated with an AMI results in perturbations in key components of MS, namely, the blood pressure, plasma glucose and serum lipid levels [4, 12–14]. In addition, medications such as beta-blockers and nitrates alter the blood pressure, and early administration of statins may alter the lipid profile, creating further confounding in the parameters used to diagnose MS. The design of the study protocol attempted to address some of these limitations. Lipid profile testing was performed the morning after admission, within 24 hours of admission, to minimize impact on serum levels. This approach is currently recommended in major guidelines [15]. Fasting glucose measurements were delayed to the morning of discharge to allow glucose metabolism to stabilize following AMI. A previous study has validated the integrity of glucose metabolism at the time of discharge and levels performed 3 months later [4]. Family doctors of all patients were contacted, and where available, fasting glucose and lipid levels obtained within the 12 months prior to the AMI were used to determine MS status.

The introduction of waist circumference as the measure of central obesity also imposes practical difficulties, as this measurement is rarely obtained during routine clinical care, in contrast to height and weight measurements, which are routinely obtained. This has led to previous studies substituting BMI for waist circumference when estimating MS prevalence utilizing data accumulated from institutional databases [16]. However, this does not estimate the true prevalence, and exposes the limitations of estimating MS prevalence from conventionally recorded data. In the present study, the prospective study design enabled targeted anthropometric measurements in all study patients, allowing for a more accurate quantification of the point prevalence of MS.

MS was more prevalent in this population than any other standard cardiac risk factor. This raises the possibility that population screening for MS may therefore identify patients at high risk for acute coronary syndromes more effectively than standard cardiac risk factors. Previous studies which have examined the prevalence of MS in the general population have reported a prevalence of 20–30% in the adult population [11, 17], compared to the prevalence of approximately 50% in the AMI cohort included in the present study. In the San Antonio Heart Study, MS according to the NCEP definition was an independent predictor of cardiovascular mortality after adjustment for age, gender, and ethnic group [2]. There was also an important association between female gender and MS by the NCEP criteria in the present study. The population included in the current study represents a high-risk population with a high-cardiovascular mortality. In the San Antonio Heart Study females with MS had nearly twice the risk for cardiovascular mortality compared to males [2]. The higher mortality risk of women compared to men with coronary disease is well recognized, and MS may well be a method to further refine risk stratification for women.

The failure of clinicians to recognize and incorporate MS status into the process of routine clinical management of patients raises further questions about the clinical validity of this syndrome. Recently, several clinicians and scientists have raised concerns about the clinical validity of this syndrome [18–21]. It is likely that controversies about the etiology of MS and its clinical validity, coupled with the practical difficulties in assessing MS status discussed above, limit clinical uptake of this syndrome in this setting. However, it must be noted that while the diagnosis of MS per se did not appear to be widely recognized clinically, its components were recognized and clinically managed as indicated, most patients were placed on statins following their AMI regardless of lipid profile; new medication for diabetes was started in 5 patients (5%); ACE-inhibitors and/or beta blockers were prescribed in the majority of patients prior to discharge.

Echocardiographic indices of diastolic function revealed greater diastolic impairment in patients with MS even though there were no differences in rates of successful reperfusion or indices of systolic function. The diastolic dysfunction is most likely the result of the higher preponderance of pre-existing hypertension/raised blood pressure and diabetes in patient with MS. Hypertensive cardiomyopathy in its various stages has a predominant impact on diastolic rather than systolic function. Similarly, diabetic patients also exhibit a degree of diastolic impairment related to microvascular dysfunction [22].

## 5. Limitations

This study has a number of key limitations. The number of patients enrolled in this prospective single center study represents a relatively small sample size. Thus the prevalence data obtained in this study should be interpreted with caution. As discussed previously, there are fundamental issues associated with the assessment of blood pressure, gluco-metabolic state, and lipid indices in the peri-myocardial infarct period. However, several design features of the study protocol attempted to limit the impact of these confounding factors. The number of women in the study was limited, which may limit the clinical significance of important gender associated differences found in the present study. The small proportion of non-Europid patients limits the generalization of results to other ethnic groups who may be more susceptible to the metabolic syndrome. Finally, while this study has focused on the prevalence and clinical management of MS in patients with AMI, the long-term prognostic implications of MS in these patients have not been examined. The lack of follow-up data with respect to hard clinical endpoints after a substantial follow-up period limits the conclusions that can be drawn from this study about the clinical significance of the MS in patients with AMI.

## Figures and Tables

**Table 1:** Baseline characteristics of patients.

Baseline Characteristic	*N* = 107
Age (years)	59 ± 13
Male	86 (80)
Ethnicity	
Europid	79 (74)
South Asian	13 (12)
Eastern Mediterranean	5 (5)
Arabic	4 (4)
Chinese	4 (4)
South American	1 (1)
Polynesian	1 (1)
Hypertension	51 (48)
Smoking	20 (19)
Diabetes mellitus	20 (19)
Family history	34 (32)
Dyslipidaemia	43 (40)
Statin on admission	19 (18)
New IFG	42 (39)
Waist Circumference (cm)	
Males	101.6 ± 12.8
Females	100.4 ± 18.3
Height (cm)	
Males	1.70 ± 0.11
Females	1.57 ± 0.09
Weight (kg)	
Males	85.0 ± 16.2
Females	72.2 ± 19.9
BMI (kg/m^2^)	
Males	29.8 ± 8.5
Female	29.4 ± 8.3
Waist: Hip	1.08 ± 0.97
Systolic BP	112.8 ± 15.1
Diastolic BP	66.9 ± 9.5
Discharge Medications	
Aspirin	91 (85)
Statin	93 (87)
Fibrate	2 (2)
Ezetimibe	3 (3)
Beta blocker	90 (84)
ACE-Inhibitor	76 (71)
ATRA	9 (8)
Clopidogrel	92 (86)

Data are presented as mean ± SD or *n* (%); BMI: body mass index; IFG: impaired fasting glucose.

**Table 2:** Characteristics of patients with and without IDF MS.

Baseline Characteristic	IDF MS (*n* = 58)	No. MS (*n* = 49)	*P* value
Age	59.7 ± 12.9	58.9 ± 12.7	NS
Male	44 (76)	42 (86)	NS
Hypertension	38 (66)	13 (27)	<.001
Smoking	20 (35)	22 (45)	NS
Diabetes mellitus	15 (26)	5 (10)	.03
Family history	20 (35)	14 (29)	NS
Dyslipidaemia	21 (36)	22 (45)	NS
New IFG	29 (50)	13 (27)	.02
Waist Circumference	107.8 ± 13.2	92.8 ± 17.9	<.001
Height	1.7 ± 0.1	1.7 ± 0.1	NS
Hip	104.1 ± 17.5	97.6 ± 16.9	.056
Weight	89.0 ± 17.4	74.9 ± 14.6	<.001
BMI	31.2 ± 5.9	27.9 ± 10.5	.048
Waist:Hip	1.18 ± 1.31	0.96 ± 0.96	NS
Systolic BP	113.8 ± 16.3	111.6 ± 13.8	NS
Diastolic BP	67.4 ± 10.8	66.3 ± 7.9	NS
Proximal LAD Disease	29 (52)	26 (54)	NS
Extent CAD			NS
1 VD	14 (25)	18 (37)	
2 VD	20 (36)	16 (33)	
3 VD	22 (38)	14 (29)	
Final TIMI 3 flow	50 (91)	42 (88)	NS
LVEF	43.7 ± 7.4	42.5 ± 8.8	NS
DT	174.0 ± 61.7	206.0 ± 58.0	.02
E′	5.2 ± 1.6	6.5 ± 2.0	.04
E to E′ ratio	13.9 ± 5.0	12.4 ± 4.8	NS
Fasting Glucose	6.8 ± 2.6	5.6 ± 0.8	.002
TC	4.8 ± 1.5	4.4 ± 0.9	NS
HDL	0.9 ± 0.3	1.1 ± 0.3	.01
LDL	3.1 ± 1.3	2.8 ± 0.8	NS
Trig	1.7 ± 0.9	1.2 ± 0.7	.001

Data are presented mean ± SD or *n* (%). BMI: body mass index; CAD: coronary artery disease; DT: deceleration time; HDL: high-density lipoprotein; IFG: impaired fasting glucose; LAD: left anterior descending artery; LDL: low-density lipoprotein; LVEF: left ventricular ejection fraction; TC: total cholesterol; VD: vessels diseased.

**Table 3:** Characteristics of patients with and without NCEP MS.

Baseline Characteristic	NCEP MS (*n* = 52)	No. MS (*n* = 55)	*P* value
Age	62.0 ± 13.5	56.8 ± 11.6	.035
Male	35 (67)	51 (93)	.001
Hypertension	37 (71)	14 (26)	<.001
Smoking	15 (29)	27 (49)	.05
Diabetes mellitus	15 (29)	5 (9)	NS
Family history	15 (29)	19 (35)	NS
Dyslipidaemia	25 (46)	18 (33)	NS
New IFG	29 (59)	13 (24)	.001
Waist Circumference	107.2 ± 15.3	94.1 ± 16.2	<.001
Height	1.67 ± 0.12	1.68 ± 0.12	NS
Hip	104.4 ± 19.3	98.0 ± 15.1	.062
Weight	87.5 ± 19.9	77.9 ± 13.8	.005
BMI	31.3 ± 6.3	28.1 ± 9.8	.056
Waist:Hip	1.2 ± 1.4	1.0 ± 0.2	NS
Systolic BP	114.0 ± 16.9	111.6 ± 13.3	NS
Diastolic BP	66.4 ± 10.8	67.3 ± 8.2	NS
Proximal LAD Disease	26 (52)	29 (53)	NS
Extent CAD			NS
1 VD	15 (30)	17 (32)	
2 VD	16 (32)	20 (37)	
3 VD	19 (38)	17 (32)	
Final TIMI 3 flow	45 (92)	47 (87)	.4
LVEF (%)	44.2 ± 7.3	42.1 ± 8.7	NS
DT	179.6 ± 65.9	197.5 ± 57.1	NS
E′	5.4 ± 1.6	6.3 ± 1.9	.046
E to E′ ratio	14.8 ± 5.5	11.8 ± 4.1	.014
Fasting Glucose	7.1 ± 2.7	5.6 ± 0.8	<.001
TC	4.9 ± 1.5	4.4 ± 1.0	.082
HDL	0.94 ± 0.29	1.06 ± 0.27	.037
LDL	3.1 ± 1.3	2.8 ± 0.8	NS
Trig	1.8 ± 0.8	1.2 ± 0.8	<.001

Data are presented mean ± SD or *n* (%). BMI: body mass index; CAD: coronary artery disease; DT: deceleration time; HDL: high-density lipoprotein; IFG: impaired fasting glucose; LAD: left anterior descending artery; LDL: low-density lipoprotein; LVEF: left ventricular ejection fraction; TC: total cholesterol; VD: vessels diseased.

**Table 4:** Multivariate predictors of metabolic syndrome.

	Odds Ratio	95% Confidence Interval	*P* value
NCEP MS			

Hypertension	955.1	9.1–100791.6	.004
Fasting Glucose	133.6	5.4–3317.7	.003
Triglycerides	8.0	1.4–45.6	.020
Waist Circumference	1.3	1.1–1.5	.004
HDL	0.003	0.000–0.256	.011
Male Gender	0.001	0.000–0.364	.024

IDF MS			

Hypertension	12.1	3.2–44.9	<0.001
Fasting Glucose	2.3	1.2–4.3	.014
Waist Circumference	1.1	1.0–1.2	.001
HDL	0.07	0.01–0.64	.018
